# Screening the Optimal Probe by Expounding the ESIPT Mechanism and Photophysical Properties in Bis-HBX with Multimodal Substitutions

**DOI:** 10.3390/molecules29112692

**Published:** 2024-06-06

**Authors:** Min Yang, Hongyan Mu, Jiaan Gao, Qi Zhen, Xiaonan Wang, Xiaotong Guan, Hui Li, Bo Li

**Affiliations:** 1 Jilin Key Laboratory of Solid-State Laser Technology and Application, School of Physics, Changchun University of Science and Technology, Changchun 130022, China; 18404361217@163.com (M.Y.); xuemuafm@163.com (H.M.); gja13622147194@163.com (J.G.); wang18338564758@163.com (X.W.); mianhuatang7657@163.com (X.G.); 2 School of Civil Engineering, Changchun Institute of Technology, Changchun 130012, China; zhenqi18@mails.jlu.edu.cn; 3 State Key Laboratory of High Power Semiconductor Lasers, School of Physics, Changchun University of Science and Technology, Changchun 130022, China

**Keywords:** intermolecular hydrogen bonding, excited-state intramolecular proton transfer, twisted intramolecular charge transfer

## Abstract

DFT and TD-DFT were used in this article to investigate the effects of different substitutions at multiple sites on the photophysical mechanism of bis-HBX in the gas phase. Four different substitution modes were selected, denoted as A_1_ (X=Me, Y=S), A_2_ (X=OMe, Y=S), B_1_ (X=Me, Y=NH), and C_1_ (X=Me, Y=O). The geometric parameters proved that the IHBs enhanced after photoexcitation, which was conducive to promote the ESIPT process. Combining the analysis of the PECs, it was revealed that the bis-HBX molecule underwent the ESIPT process, and the ease of the ESIPT process was in the order of A_1_ > A_2_> B_1_ > C_1_. In particular, the TICT process in A_1_ and B_1_ promoted the occurrence of the ESIPT process. In addition, the IC process was identified, particularly in C_1_. Meanwhile, the calculation of fluorescence lifetime and fluorescence rate further confirmed that A_1_ was the most effective fluorescent probe molecule. This theoretical research provides an innovative theoretical reference for regulating ESIPT reactions and optimizing fluorescent probe molecules.

## 1. Introduction

**Scheme 1:**
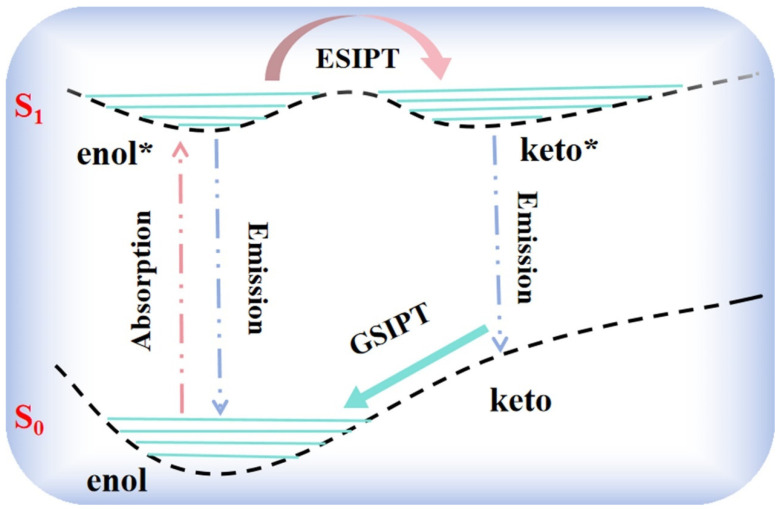
Schematic diagram of ESIPT reaction.

The excited-state intramolecular proton transfer (ESIPT) process is a photo-induced enol-keto tautomerization process [1,2,3] accompanied by strong intramolecular hydrogen bonds (IHBs) [4,5], which allows proton H from specific amino or hydroxyl atoms to transfer to carbonyl oxygen or azo nitrogen atoms after photoexcitation. As shown in Scheme 1, the integral ESIPT reaction is essentially a four-level photo-cycle process. Firstly, the enol form in the ground state is photoexcited to the Franck–Condon point of the S_1_ state, generating the enol* form simultaneously. Secondly, the enol* form undergoes the ESIPT process to produce a tautomer keto (keto*) form. Thirdly, the keto* form relaxes back to the ground-state keto structure via releasing the fluorescence or non-radiative transition. Eventually, the keto form is converted to the initial thermodynamically stable enol form achieved by the reverse proton transfer (RPT) process. Generally speaking, the ESIPT system tends to exhibit a large Stokes shift and dual fluorescence [6,7,8]. In the 1950s, Weller first observed the double fluorescence phenomenon during an experimental study on the dynamic characteristics of methyl salicylate and attributed it to the ESIPT reaction [9]. Since then, the mechanism of the ESIPT reaction has attracted the attention of extensive researchers, most of whom have studied it experimentally and theoretically [10,11,12,13].

2-(2′-hydroxyphenyl) benzazole (HBX) derivatives with ESIPT properties are typically characterized by high yield and low synthesis complexity [14]. This distinctive nature makes them widely used in laser dyes [15], chemical sensors [16,17], molecular switches [18], light-emitting diode devices [19], and fluorescent probes for use in biological systems [20,21]. Previous reports have shown that modifying the molecular structure can effectively regulate the ESIPT process of HBX derivatives. In 2017, Manojai et al. [22] investigated the effect of heteroatom substitution (X=NH, O, and S) on the photophysical properties of HBX derivatives. They proved that NH substitution increased the probability of the ESIPT process in HBT, resulting in redshift emission fluorescence. In 2020, yang et al. [23] probed the effects of -NH_2_ group substitutions at different positions on the ESIPT mechanism. They revealed that the para-substitution of the -NH_2_ group promoted the occurrence of the ESIPT process. In 2022, Zhang et al. [24] designed four molecules (BPN, BPNS, BPS, and BPSN) to explore the effects of different electron groups on the ESIPT process in depth. They believed that introducing electron-withdrawing groups accelerates the ESIPT process, while the electron-donating groups do the opposite. It should not be ignored that in previous studies on HBX derivatives, researchers mainly focused on the effects of substitutions on the mono-HBX [22,23,24,25]. Recently, Lee et al. [26] designed and synthesized bis 2-(2′-hydroxyphenyl) benzazole (bis-HBX) fluorescent probe molecules based on two HBX moieties. The bis-HBX had extra proton-binding sites and was more effective for the detection of alkaline pH than mono-HBX [26,27]. However, the internal mechanism of the effects of different substitutions at multiple sites on the fluorescence properties of bis-HBX was still deficient, and the fluorescence probe molecules with the strongest luminous efficiency were screened, which merits further attention. Therefore, a thorough theoretical investigation was urgently needed to better understand the ESIPT process and the effects of different substitutions at multiple sites on the fluorescence properties of bis-HBX.

**Scheme 2:**
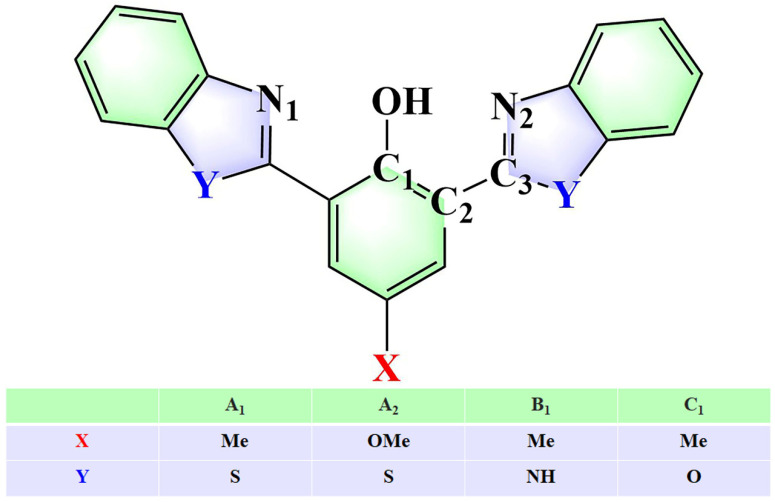
Chemical configuration diagram of bis-HBX.

In this study, we simultaneously adjusted two key parameters, namely the extension group of central phenol (X: Me or OMe) and the properties of heteroatoms (Y: S, O, N) (as shown in Scheme 2). Using DFT and TD-DFT methods, the effects of different substitutions at multiple sites on the photophysical mechanism and the ESIPT process in bis-HBX were rationally researched. Our research not only provides a new idea and method for improving the efficiency of the ESIPT process but also has an important reference value for designing and optimizing fluorescent probe molecules with high fluorescence efficiency.

## 2. Results and Discussion

### 2.1. Geometric Parameters of Bis-HBX

**Figure 1:**
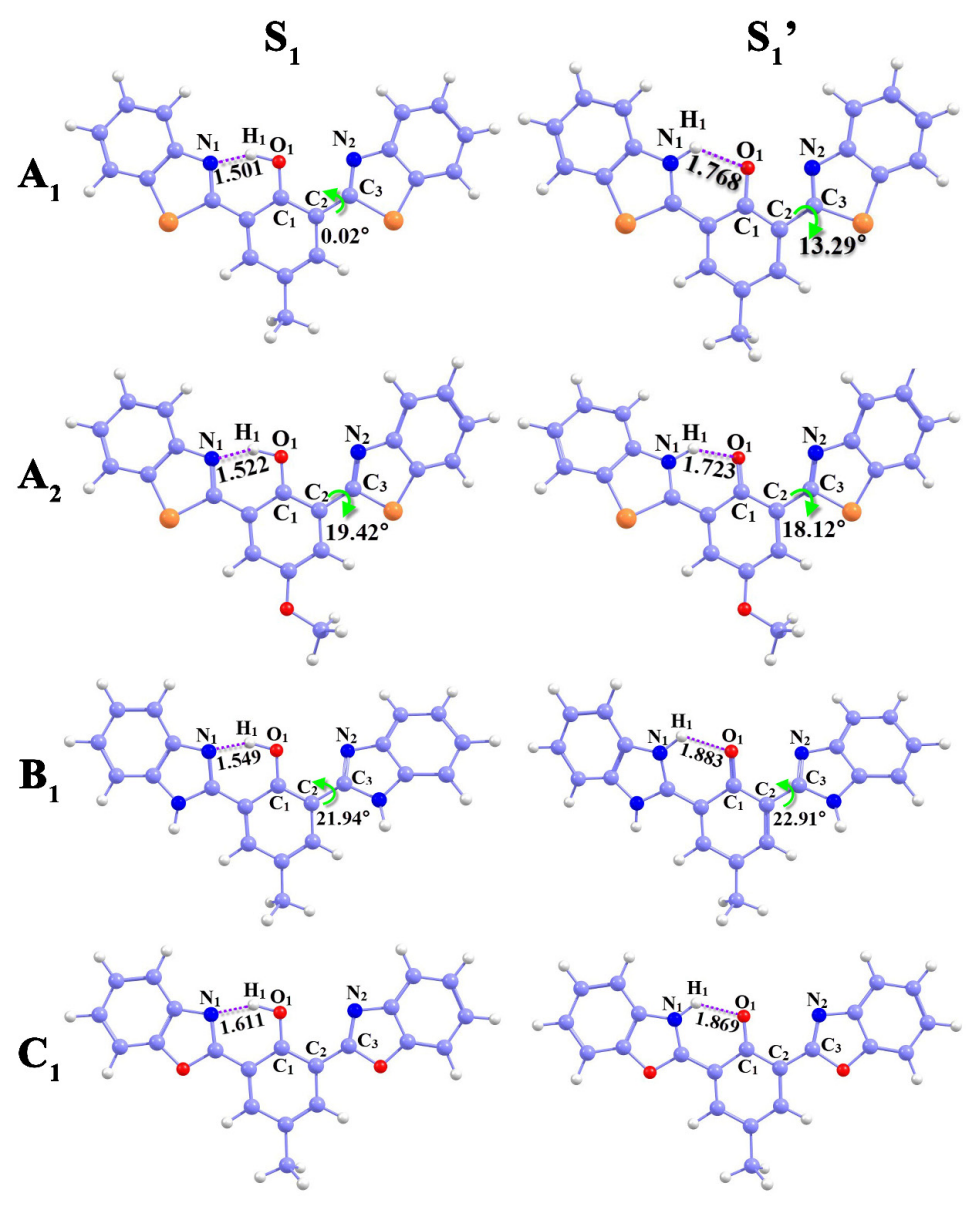
Optimized geometry of bis-HBX in different states.

All the geometric configurations of different states of bis-HBX (see Figure 1 and Figure S1) were optimized completely by the B3LYP-D3/6-31G+(d,p) method without any restrictions on bond lengths or bond angles. In the excited state, we labeled the enol* structure as the S_1_ state and the keto* structure as the S_1_′ state. Some of the significant geometric parameters are displayed in Table 1, including bond lengths and bond angles.

The intramolecular hydrogen bonding (IHB) parameters of bis-HBX from the S_0_ to the S_1_ state followed the similar varying tendency recorded in Table 1. We chose A_1_ as a representative for ease of expounding and fully discussing the changing trend in IHB intensity upon photoexcitation. The bond length (O_1_–H_1_) of the A_1_ molecule was elongated from 0.994 Å (S_0_) to 1.058 Å (S_1_), and N_1_-H_1_ was decreased from 1.720 Å (S_0_) to 1.501 Å (S_1_). Furthermore, the bond angle δ (O_1_-H_1_-N_1_) was increased from 147.14° to 153.08°. It has been validated that the IHB (O_1_-H_1_…N_1_) intensity is enhanced in the S_1_ state, which is conducive to the ESIPT process [28,29,30]. In addition, in the S_1_′ state, we obtained tautomer configuration, and a new IHB (N_1_-H_1_…O_1_) was formed with a bond length of 1.036 Å. The bis-HBX molecule underwent the ESIPT process, which was proved by the above-calculated results.

It is noteworthy that, in contrast to C_1_, the dihedral angle δ (C_1_–C_2_–C_3_–N_2_) of A_1_, A_2,_ and B_1_ existed a torsion between the central benzene ring and the right five-membered ring. The δ (C_1_–C_2_–C_3_–N_2_) of A_1_, called the dihedral angle, enlarged from 47.02°(S_0_) to 13.29° (S_1_′), with a torsion of 60.31°. Similarly, both the A_2_ and B_1_ molecules underwent torsions of 28.16° and 24.69°, respectively. All the alterations indicated that A_1_, A_2_, and B_1_ underwent the ESIPT process with distortions in molecular configuration.

### 2.2. Infrared (IR) Vibrational Spectra

Due to the IR vibrational spectra providing direct evidence for the formation and fracture of hydrogen bonds (HBs) during the ESIPT process [31], we simulated the IR vibrational spectra of four probes in different electronic states.

**Figure 2:**
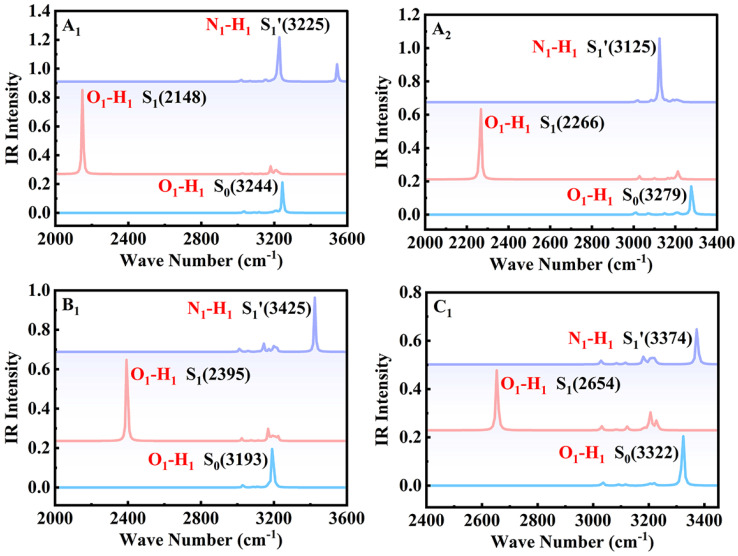
Simulated IR spectra of O_1_–H_1_ and N_1_–H_1_ stretching vibration frequencies of bis-HBX in different electronic states in the gas phase.

From the simulated IR spectra (Figure 2), we found that the stretching vibration frequencies of O_1_-H_1_ in A_1_, A_2_, B_1_, and C_1_ were separately redshifted from 3244 cm^−1^, 3279 cm^−1^, 3193 cm^−1^, and 3322 cm^−1^ in the S_0_ state to 2148 cm^−1^, 2266 cm^−1^, 2359 cm^−1^, and 2654 cm^−1^ in the S_1_ state, accompanied with redshifts of 1096 cm^−1^, 1013 cm^−1^, 798 cm^−1^, and 668 cm^−1^, respectively. The above data indicated that the redshift value followed the order: A_1_ > A_2_ > B_1_ > C_1_, as we all know the weakening and strengthening of the IHB can be explained by the blue- and redshifts in the IR vibrational frequencies of the O-H bond [32,33,34], respectively, so we can infer that the ESIPT process was more prone to occur in A_1_. Meanwhile, in the S_1_′ state, a new vibrational peak was observed, corresponding to the stretching vibration frequency of N_1_–H_1_, which was located around 3225 cm^−1^ (A_1_), 3125 cm^−1^ (A_2_), 3425 cm^−1^ (B_1_), and 3374 cm^−1^ (C_1_). The above discussion confirmed that the four probe molecules underwent the ESIPT process after photoexcitation.

### 2.3. RDG Scatter Plot and Topological Analyses

Considering that using the scatter plot of RDG versus Sign(I_2_)r can effectively reveal IHB interactions in real space [35], and Lu et al. recently proposed the IRI isosurface based on RDG versus Sign(I_2_)r, the expression of RDG function was as shown in Equation (1) [36,37]:

(1) $$RDG\left(r\right)=\frac{1\left|\nabla \rho \left(r\right)\right|}{2{\left(3\pi^{2}\right)}^{1/3}\rho {\left(r\right)}^{\frac{4}{3}}}$$

The obtained electron density matrix equation was Equation (2):

(2) $$\Omega \left(r\right)=sign\left(I_{2}\left(r\right)\right)\rho \left(r\right)$$

**Figure 3:**
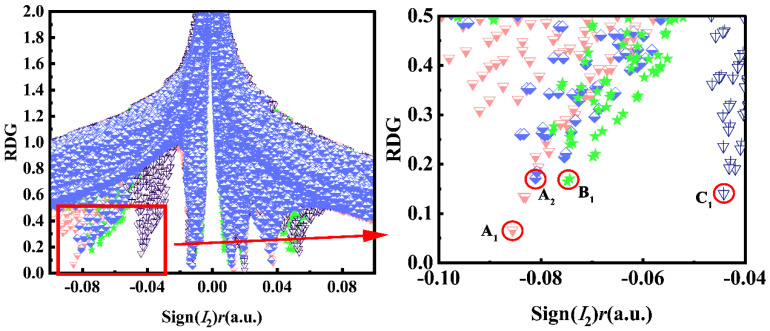
Sign(I_2_)r scatter plots versus reduced density gradient (RDG) in bis-HBX in the S_1_ state.

As clarified in Figures S2 and S3, the blue section of the color level corresponds to the HB effect, and the intensity of HB gradually increased with the deepening of the color. In addition, the spatial interactions and van der Waals forces were represented by the red and green areas, respectively. Through analyzing the RDG versus Sign(I_2_)r scatter plot, the relationship of IHB intensity in A_1_, A_2_, B_1_, and C_1_ was clear-cut and unambiguous. As shown in Figure 3, with the substitution modes of C_1_, B_1_, A_2_, and A_1_, the Sign(I_2_)r values became more and more negative, unveiling that the strength of IHB in different surroundings obeys the order of A_1_ > A_2_ > B_1_ > C_1_. In the S_0_ state, the prong peaks of A_1_, A_2_, B_1_, and C_1_ were located at −0.050, −0.049, −0.050, and −0.044, respectively. After photoexcitation, the prong peaks of A_1_, A_2_, B_1_, and C_1_ shifted to the more negative region (−0.087, −0.081, −0.075, and −0.063, respectively). The interaction type was judged to be an IHB interaction, corresponding to O_1_–H_1_…N_1_. The IHB intensity of the four compounds was enhanced after photoexcitation, as indicated by the above-mentioned data, which is conducive to the ESIPT process. Moreover, comparison of the left-shifted peak positions in the four compounds revealed that the strength of IHB followed the order of A_1_ > A_2_ > B_1_ > C_1_ in the S_1_ state. This was consistent with our previous analysis conclusion, revealing that different substitutions at multiple sites did affect the ESIPT process.

Topological analysis, first proposed by Bader [38], is also one of the most common methods for measuring the strength of HBs [39]. The obvious bond paths and bond critical points (BCPs) [40] in the IHB region are captured in Figure S4, and we have listed the relevant parameters in Table 2. As is known to all, ρ(r) and ν(r) can gauge the intensity of IHBs with advantages, while the larger ρ(r) is and the more negative ν(r) is, the stronger IHB intensity will be. It could clearly be seen that the IHBs of the four molecules were all enhanced in the S_1_ state. Moreover, a horizontal comparison of the absolute values of *E*_HB_ in bis-HBX showed that the *E*_HB_ values followed the order of *E*_HB_(BCP2) > *E*_HB_(BCP4) > *E*_HB_(BCP6) > *E*_HB_(BCP8), indicating that the IHB intensity of A_1_ was the strongest of the four molecules in the S_1_ state. It is well known that the stronger the IHB intensity is, the more favorable the ESIPT process is. Therefore, through the analysis of the above methods, we believe that the stronger the IHBs interaction, the easier the ESIPT process.

### 2.4. Fluorescence Attribution and Mechanism Analysis

**Figure 4:**
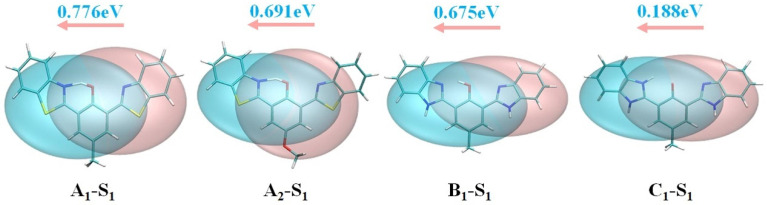
Hole–electron analysis in the S_1_′ state of bis-HBX (the blue isosurface represents the distribution of electrons, and the pink isosurface represents the distribution of holes).

We performed electron–hole analysis on bis-HBX molecules in the S_1_ state, as shown in Figure 4. Furthermore, they were intuitively analyzed by utilizing the inter-fragment charge transfer (IFCT) method. During the emission process, from the right side to the central benzene ring direction, 0.776 electrons, 0.691 electrons, 0.675 electrons, and 0.188 electrons were transmitted, respectively. Moreover, the characteristics of hole and electron distribution indexes are included in Table 3. It was well known that for analogs, the lower the Sr index and the more positive the t index, the more complete the separation of electrons and holes. In the light of the t and Sr indexes, a significant separation of holes and electrons was implied through the S_0_→S_1_ transition in A_1_ and B_1_, which proved that A_1_ and B_1_ had significant ICT (intramolecular charge transfer) characteristics. Furthermore, combined with the configuration of bis-HBX, the dihedral angles δ (C_1_–C_2_–C_3_–N_2_) of A_1_ and B_1_ had abnormal twists of 47 degrees and 24.69 degrees, respectively, during photoexcitation. Perspicuously, it can be confirmed that the typical twisted intramolecular charge transfer (TICT) [41,42] process existed in A_1_ and B_1_. From the above analysis results, it can be seen that A_1_ and B_1_ first underwent the TICT process in the S_1_ state, and then underwent the ESIPT process. Thus, we know that the TICT process in A_1_ and B_1_ facilitated the ESIPT process.

**Figure 5:**
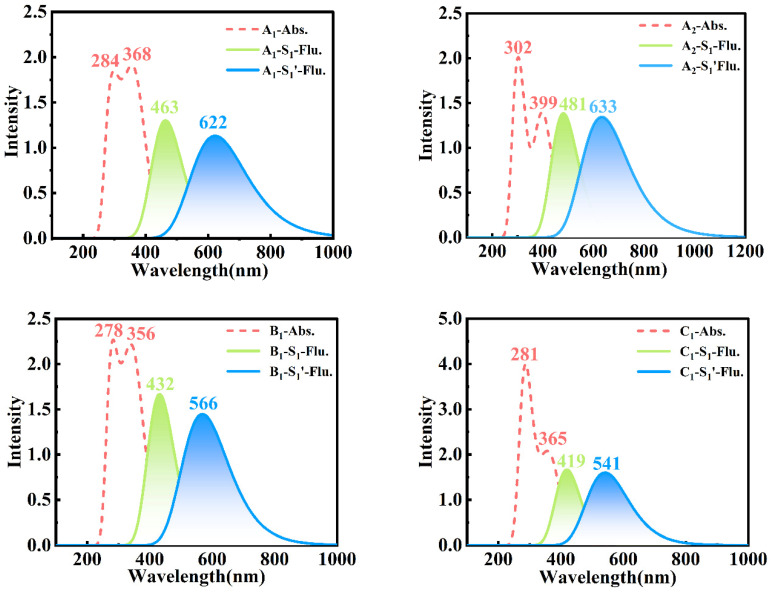
Simulations of the absorption and fluorescence spectra of bis-HBX (in the gas phase) via the B3LYP-D3/6-31+G (d, p) method.

In order to gain a deeper understanding of the effects of different substitutions at multiple sites on photophysical properties in bis-HBX, we simulated the absorption and fluorescence spectra of bis-HBX (see Figure 5), which were obtained based on the optimized S_0_, S_1_, and S_1_′ configurations. As shown in Table 4, the relevant photophysical parameters were recorded, and the absorption peak, oscillator strength, and corresponding orbital transition contributions were also included.

The bis-HBX had double absorption peaks that could be distinctly observed from the data listed in Table S1, in which the calculated maximum absorption peak was very consistent with the experimental value, proving the feasibility of our method [26]. In addition, as shown in Figure 5, the short-wavelength emission peaks of A_2_, A_1_, B_1_, and C_1_ were 481 nm, 463 nm, 432 nm, and 419 nm, showing a blueshift from A_2_ to C_1_, respectively. The proton transfer tautomers in A_2_, A_1_, B_1_, and C_1_ corresponding to the longer wavelength fluorescence peaks were 633 nm, 622 nm, 566 nm, and 541 nm, respectively. Obviously, with the introduction of different substitution forms, the significant redshift was produced in the longer wavelength emission peaks of A_2_, A_1_, B_1_, and C_1_, and the degree of redshift followed the order of A_1_ > A_2_ > B_1_ > C_1_. However, only one short-wavelength fluorescence was observed in A_1_ in accord with the emission peak in the experiment; thus, we attributed it to the S_1_ (TICT) state.

Moreover, in order to study the fluorescence properties in bis-HBX more comprehensively, we used Equations (3) and (4) [36,37]:

(3) $$\tau =\frac{1.499}{f_{1}E^{2}}\times 10^{-5}$$

(4) $$f_{2}=\frac{1}{\tau }$$

We calculated the fluorescence lifetime and rate in the bis-HBX and listed them in Table 5. In the formula, $f_{1}$ represents the oscillator strength and E delegates the wavenumber. It can be observed that A_1_ exhibited the greatest fluorescence lifetime, which may be due to its lower oscillation intensity. Similarly, a larger fluorescence lifetime resulted in a lower fluorescence rate, with A_1_ exhibiting the lowest fluorescence rate. All the above analyses indicated that A_1_ was the fluorescence probe molecule with the strongest luminous efficiently in bis-HBX, and we were also able to confirm that different substitutions at multiple sites did affect the spectral characteristics of bis-HBX.

**Figure 6:**
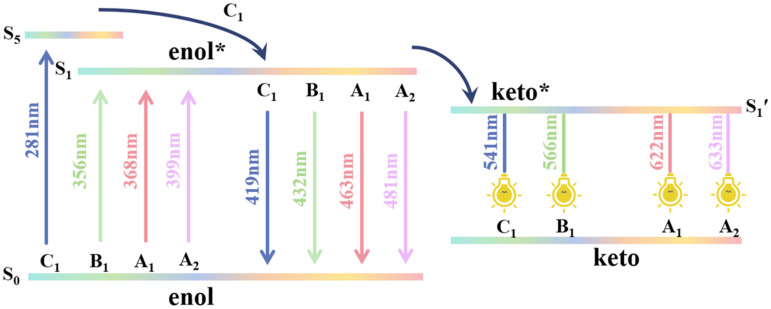
Abridged sketch of the excitation process in bis-HBX.

Finally, we conducted a detailed analysis of the excited-state decay process in bis-HBX. We have provided a diagrammatic sketch of the excitation and emission processes in bis-HBX (Figure 6) according to Table 4. Perspicuously, from the oscillator strength in Table 3, the excited states of A_1_, A_2_, and B_1_ were separated from each other. It was interesting that the excited states of S_1_ (0.4634) and S_5_ (0.4930) in C_1_ were coupled together and formed an intersection point where the internal conversion (IC) took place. The vertical transition S_1_→S_0_ that occurred at the Franck–Condon region of A_1_, A_2_, B_1_, and C_1_ was accompanied by shorter wavelength fluorescence measurements of 463 nm, 481 nm, 432 nm, and 419 nm, respectively. Subsequently, in the S_1_ state, from the higher vibration level to the lowest vibration level, a vibration relaxation process occurred in which A_1_, A_2_, B_1_, and C_1_ recovered from the tautomer structure to the S_0_ state, while at 622 nm, 633 nm, 566 nm, and 541 nm, respectively, they emitted long-wavelength fluorescence.

### 2.5. Frontier Molecular Orbitals and NBO Population

**Figure 7:**
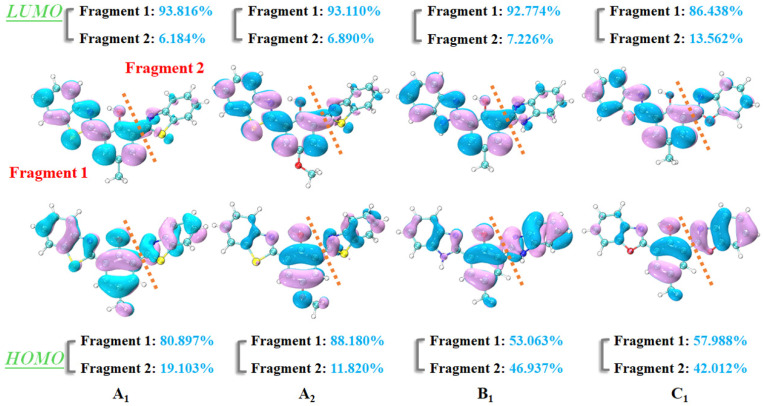
Frontier molecular orbitals of bis-HBX in the gas phase.

It is particularly acknowledged that frontier molecular orbitals (FMOs) are an effective means to reflect charge distribution and recombination [43]. Since the first single transition of the target compound is mainly related to the highest occupied orbital (HOMO) and the lowest unoccupied orbital (LUMO), Figure 7 only renders these two orbitals. During the photoexcitation process, the electronic clouds on the O_1_ and N_1_ atoms were drastically reduced and increased, respectively. This result indicated that there was a strong binding ability between the N_1_ atom and the H_1_ proton, which provided the driving force for the ESIPT process. For the purpose of studying the changes in electron density distribution on O_1_ and N_1_ quantitatively, the NBO charge distributions of bis-HBX are recorded in Table 6. The negative charges on the O_1_ shrank from −0.693 a. u., −0.707 a. u., −0.693 a. u., and −0.696 a. u. in the ground state to −0.677 a. u., −0.685 a. u., −0.684 a. u., and −0.676 a. u. in the excited state, while that of the N_1_ atom was enlarged during the photo-absorption process, which was confirmed by the analytical results of the FMOs.

In addition, we used the Hirshfeld method to calculate the electron density components in Fragment 1 and Fragment 2 in bis-HBX (see Figure 7). In Fragment 1, the electron density components of A_1_, A_2,_ and B_1_ increased from 80.897%, 88.180%, 53.063%, and 57.988% in HOMO to 93.816%, 93.110%, 92.774%, and 86.433% in LUMO, respectively. Regarding Fragment 2, the electron density components were 19.103%, 11.820%, 46.937%, and 42.012% in HOMO, while they separately decreased to 6.184%, 6.890%, 7.226%, and 13.562% in LUMO. Meanwhile, as mentioned in the geometric parameters, the obtained dihedral angles δ (C_1_–C_2_–C_3_–N_2_) of A_1_, A_2_, B_1,_ and C_1_ underwent torsions of 47°, 28.16°, 24.69°, and 0°, respectively, which demonstrated that A_1_ has obvious TICT characteristics.

### 2.6. Potential Energy Curves (PECs)

With the aim of uncovering the mechanism of the ESIPT process with different substitutions at multiple sites more intuitively, the potential energy curves (PECs) in bis-HBX were scanned via prolonging the O_1_–H_1_ bond length from 0.9 Å to 1.9 Å in increments of 0.1 Å based on the optimized structures in the gas phase [44].

**Figure 8:**
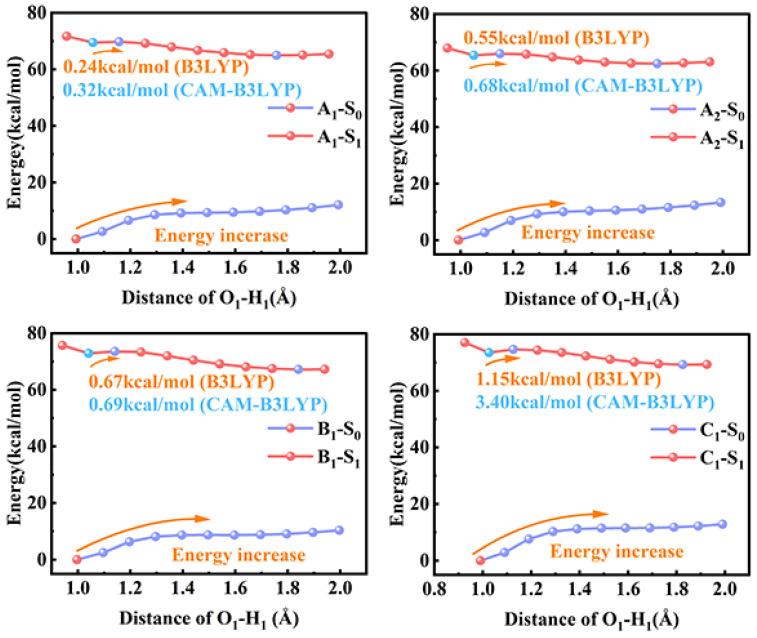
The PECs of bis-HBX in different states.

As shown in Figure 8, the PECs of the four probe molecules showed an ascending trend in the S_0_ state. This demonstrates that it was difficult for the proton transfer (PT) process to occur. After photoexcitation, the energy barriers in the four probe molecules were diminished to 0.24 kcal/mol (A_1_), 0.55 kcal/mol (A_2_), 0.67 kcal/mol (B_1_), and 1.15 kcal/mol (C_1_), respectively. It is obvious that it was more possible for the four probe molecules to undergo the PT process in the S_1_ state. It is a remarkable fact that the order of energy barriers in bis-HBX followed the order of A_1_ < A_2_ < B_1_ < C_1_, which verified that A_1_ can occur the ESIPT process more easily. Although the B3LYP/6-31+G (d,p) can provide qualitative descriptions of the hypersurface, it may underestimate some of the energy differences and energy barriers. We recalculated the PECs using Cam-B3LYP/TZVP [45,46] theory (see Figure 8; the blue font is the corrected energy barrier), taking the corrected results as the reference. It was very clear and concise that the energy barriers calculated using Cam-B3LYP/TZVP theory were consistent with the order of A_1_ < A_2_ < B_1_ < C_1_. In addition, to further confirm the ESIPT process mechanism, we also performed transition-state (TS) calculations and obtained the intrinsic reaction coordinate (IRC) to validate the reasonableness and accuracy. Meanwhile, we have listed the ESIPT barriers via the IRC paths for bis-HBX in Table 7, which is consistent with the simulation results of the PECs. We further confirmed that A_1_ can cause the ESIPT process to occur more easily. To date, we have explained the effects of different substitutions at multiple sites in the ESIPT process of bis-HBX molecules and reasonably elucidated the whole kinetic process of the studied molecules. This provides important reference values for the application of fluorescent probe molecules.

## 3. Calculation Details

In the gas phase, the geometric structures of different electronic states of bis-HBX were fully optimized by DFT [46,47,48,49,50,51] and TD-DFT [52,53,54,55] based on B3LYP-D3/6-31+g (d, p) [47,48] levels. We chose six different functionals to calculate the absorption spectra of bis-HBX, and the results are listed in Table 8. The calculated values of B3LYP-D3 (295 nm and 368 nm) were compatible with the experiment values (290 nm and 363 nm) [26]. Therefore, it was reasonable to use the B3LYP-D3 functional to evaluate the excited-state properties of the system. Based on the optimized geometric configurations, we calculated the infrared (IR) [56] vibration spectra and confirmed that all the local minima had no virtual frequency. For the purpose of probing into the study of the ESIPT mechanism in bis-HBX, the PECs (potential energy curves) of bis-HBX were scanned by setting O_1_-H_1_ from 0.9 Å to 1.9 Å in steps of 0.1 Å. The transition-state (TS) structure was located by the Berny algorithm to obtain a more accurate barrier height [57]. In addition, the RDG scatter plot [35], electron–hole, and frontier molecular orbitals (FMOs) [58,59] were analyzed by using the Multiwfn program and visualized by the VMD 1.9.4 software [60,61]. All calculation details of the system were carried out by the Gaussian program [62].

## 4. Conclusions

In summary, the DFT/TDDFT theoretical method was used to systematically investigate the effects of different substitutions at multiple sites on the photophysical properties of bis-HBX. Relying on the results of IR vibration spectra, RDG scatter plots, and topological structure, it was demonstrated that the IHBs of bis-HBX were enhanced in the S_1_ state and underwent the ESIPT process in the S_1_′ state. Significantly, upon analyzing the energy barrier height of bis-HBX, we noticed that the ease of the ESIPT process followed the order of A_1_ > A_2_ > B_1_ > C_1_. Furthermore, combining molecular configuration and electron–hole analysis, it was demonstrated that A_1_ and B_1_ underwent severe twisting (TICT state), which promoted the ESIPT process. Meanwhile, the internal conversion process was verified in C_1_. Finally, it was verified that A_1_ is the most suitable fluorescence probe molecule for detecting alkaline pH values when combined with the calculated fluorescence lifetime and fluorescence rate. Herein, the above research not only proposed the photophysical mechanism of bis-HBX, but also provided the effects of different substitutions at multiple sites on the ESIPT process in bis-HBX. Our work has provided strong support for the experiment, and in the meantime, we are sincerely anxious for this work to shed some light on regulating the photophysical behaviors and ESIPT reactions of developing and designing fluorescence probe molecules at the theoretical level.

## Figures and Tables

**Table 1:** Calculated bond lengths (Å), bond angles (°), and dihedral angles (°) of bis-HBX in different states in the gas phase.

		O_1_–H_1_	H_1_–N_1_	δ (O_1_–H_1_–N_1_)	δ (C_1_–C_2_–C_3_–N_2_)
A_1_	S_0_	0.994	1.720	147.14	−47.02
	S_1_	1.058	1.501	153.08	−0.02
	S_1_′	1.768	1.036	134.02	13.29
A_2_	S_0_	0.992	1.727	147.08	46.28
	S_1_	1.050	1.522	152.73	19.42
	S_1_′	1.723	1.043	135.84	18.12
B_1_	S_0_	0.995	1.712	147.53	−47.60
	S_1_	1.041	1.549	151.92	−21.94
	S_1_′	1.883	1.024	127.12	−22.91
C_1_	S_0_	0.990	1.764	146.80	0.12
	S_1_	1.026	1.611	150.90	0.01
	S_1_′	1.869	1.028	127.24	−0.02

**Table 2:** Calculated BCP parameters of bis-HBX. a: density of all electrons; b: potential energy density; c: Laplacian of electron density; d: Lagrangian kinetic energy; e: energy density; f: hydrogen bond energy E_HB_ = V(r)/2.

		*ρ*(*r*) ^a^ (a.u.)	*ν*(*r*) ^b^ (a.u.)	∇^2^(*ρ*) ^c^ (a.u.)	G(r) ^d^ (a.u.)	H(r) ^e^ (a.u.)	E_HB_^f^ (kcal/mol)
A_1_	BCP1	0.050	−0.038	0.121	0.034	−0.004	−11.986
	BCP2	0.086	−0.085	0.105	0.056	−0.029	−26.795
A_2_	BCP3	0.049	−0.037	0.120	0.034	−0.004	−11.735
	BCP4	0.081	−0.078	0.115	0.053	−0.025	−24.473
B_1_	BCP5	0.050	−0.039	0.121	0.034	−0.004	−12.111
	BCP6	0.075	−0.069	0.123	0.050	−0.019	−21.712
C_1_	BCP7	0.044	−0.033	0.112	0.030	−0.002	−10.291
	BCP8	0.044	−0.033	0.112	0.030	−0.002	−10.291

**Table 3:** Indexes illustrating the distribution of holes and electrons in bis-HBX. Sr: the extent of hole-and-electron overlap; t: the separation of electrons and holes.

	A_1_–S_1_	A_2_–S_1_	B_1_–S_1_	C_1_–S_1_
Sr	0.554	0.609	0.556	0.587
D (Å)	3.551	2.420	3.775	2.627
H (Å)	3.423	3.361	3.443	3.538
t (Å)	0.795	−0.182	0.945	−0.340

**Table 4:** Calculated absorption peak (λ), oscillator intensity (ƒ), and orbital contribution (%) of bis-HBX in the gas phase.

	Transition	$\lambda_{abs}$	ƒ	Composition	CI (%)
A_1_	S_0_→S_1_	368	0.3971	H→L	69.51%
	S_0_→S_2_	323	0.0761	H-2→L	49.83%
	S_0_→S_3_	320	0.1709	H-2→L	49.07%
	S_0_→S_4_	298	0.0101	H-3→L	56.49%
	S_0_→S_5_	295	0.0623	H→L+1	59.81%
	S_0_→S_6_	284	0.2971	H-4→L	60.31%
A_2_	S_0_→S_1_	399	0.3376	H→L	70.02%
	S_0_→S_2_	324	0.0475	H-2→L	50.33%
	S_0_→S_3_	321	0.1545	H-2→L	47.17%
	S_0_→S_4_	317	0.0404	H→L+1	64.43%
	S_0_→S_5_	302	0.0522	H-3→L	56.66%
	S_0_→S_6_	292	0.3057	H-4→L	55.06%
B_1_	S_0_→S_1_	356	0.4091	H→L	69.77%
	S_0_→S_2_	319	0.1957	H-1→L	62.17%
	S_0_→S_3_	312	0.1080	H-2→L	62.75%
	S_0_→S_4_	283	0.0101	H-3→L	59.64%
	S_0_→S_5_	278	0.3668	H-4→L	52.07%
	S_0_→S_6_	277	0.1199	H→L+1	63.10%
C_1_	S_0_→S_1_	365	0.4634	H→L	69.14%
	S_0_→S_2_	313	0.2566	H-1→L	68.58%
	S_0_→S_3_	296	0.0475	H-2→L	64.08%
	S_0_→S_4_	288	0.3273	H→L+1	62.92%
	S_0_→S_5_	281	0.4930	H-1→L+1	60.23%
	S_0_→S_6_	272	0.0478	H-3→L	52.42%

**Table 5:** Photophysical parameters of bis-HBX. τ represents fluorescence lifetime (ns); $f_{2}$ represents fluorescence rate (×10^−8^).

	Transition	$\lambda_{abs}$	ƒ_1_	Composition	CI (%)	τ	$f_{2}$
A_1_	S_0_→S_1_	368	0.3971	H→L	69.51%	5.1121	0.1956
A_2_	S_0_→S_1_	399	0.3376	H→L	70.02%	5.1027	0.1960
B_1_	S_0_→S_1_	356	0.4091	H→L	69.77%	4.6438	0.2240
C_1_	S_0_→S_5_	281	0.4930	H-1→L+1	60.23%	2.4009	0.4165

**Table 6:** NBO charge allotments (a. u.) of the O_1_ and N_1_ in bis-HBX in different states.

	A_1_		A_2_		B_1_		C_1_	
	S_0_	S_1_	S_0_	S_1_	S_0_	S_1_	S_0_	S_1_
O_1_	−0.693	−0.677	−0.707	−0.685	−0.693	−0.684	−0.696	−0.676
N_1_	−0.515	−0.523	−0.514	−0.519	−0.543	−0.545	−0.540	−0.547

**Table 7:** Theoretical PT barriers (kcal/mol) calculated from the PECs (B3LYP and Cam-B3LYP) and from IRCs (S_1_ state) for bis-HBX.

	A_1_	A_2_	B_1_	C_1_
B3LYP	0.24	0.55	0.67	1.15
Cam-B3LYP	0.32	0.68	0.69	3.40
IRC	0.35	0.67	0.69	3.42

**Table 8:** Using six functionals, we calculated absorption peaks (nm) in bis-HBX. (Exp. was the experimental value.)

Exp.	B3LYP [63]	B3LYP-D3 [47]	Cam-B3LYP [64]	M06-2X [65]	MPW1PW91 [66]
290	295	295	251	250	284
363	370	368	321	320	357

## Data Availability

The original contributions presented in the study are included in the article and Supplementary Material.

## Supplementary Materials

The following supporting information can be downloaded at: https://www.mdpi.com/article/10.3390/molecules29112692/s1, Figure S1: Optimized geometry of bis-HBX in ground state; Table S1: Experimental and theoretical values of absorption and fluorescence peaks of bis-HBX; Figure S2: Sign(I2)r scatter plots versus reduced density gradient (RDG) in A1 and A2 in the different states; Figure S3: Sign(I2)r scatter plots versus reduced density gradient (RDG) in B1 and C1 in the different states; Figure S4: Topological diagrams of bis-HBX. Ground state optimized Cartesian coordinates.

## References

[1] Yang X., Yang Z., Li H., Yang X., Zhang Y. (2023). Effect of Pyridyl Position on Constructing Acid-Base Dual-Responsive Fluorophores with ESIPT and AIE Characteristics. Dyes Pigment..

[2] Stoerkler T., Laurent A.D., Ulrich G., Jacquemin D., Massue J. (2023). Influence of Ethynyl Extension on the Dual-State Emission Properties of Pyridinium-Substituted ESIPT Fluorophores. Dyes Pigment..

[3] Pan Q., Jia D., Zhang Y., Ding Y. (2023). A Butterfly-Shaped ESIPT Dye for Pattern Recognition of Metal Ions. Dyes Pigment..

[4] Zahid Nasim S., Sarfaraz S., Jan F., Yar M., Ur Rehaman A. (2023). Computational Insights of Excited State Intramolecular Proton Transfer (ESIPT) Based Fluorescent Detection and Imaging of γ-Glutam-ytranspeptidase Activity. Spectrochim. Acta A Mol. Biomol. Spectrosc..

[5] Pang H., Lilla E.A., Zhang P., Zhang D., Shields T.P., Scott L.G., Yang W., Yokoyama K. (2020). Mechanism of Rate Acceleration of Radical C-C Bond Formation Reaction by a Radical SAM GTP 3′,8-Cyclase. J. Am. Chem. Soc..

[6] Mu H., Sun Y., Gao J., Xin C., Zhao H., Jin G., Li H. (2023). Switching the ESIPT and TICT Process of DP-HPPI via Intermolecular Hydrogen Bonding. J. Mol. Struct..

[7] Li H., Mu H., Xin C., Cai J., Yuan B., Jin G. (2022). Turning ON/OFF the Fluorescence of the ESIPT State by Changing the Hydrogen Bond Distance and Orientation in Quinoline-Pyrazole Derivatives. J. Mol. Struct..

[8] Gao J., Mu H., Zhen Q., Guan X., Li H. (2023). Accelerating the Concerted Double Proton Transfer Process of 2,2′-Bipyridine-3,3′-Diol-5,5′-Dicarboxylate Acid Ethyl Ester (BPDC) Molecule by Centrosymmetric Dual Intermolecular Hydrogen Bonds. J. Mol. Struct..

[9] Pariat T., Munch M., Durko-Maciag M., Mysliwiec J., Retailleau P., Vérité P.M., Jacquemin D., Massue J., Ulrich G. (2021). Impact of Heteroatom Substitution on Dual-State Emissive Rigidified 2-(2′-hydroxyphenyl)Ben-zazole Dyes: Towards Ultra-Bright ESIPT Fluoro-phores. Chem.-Eur. J..

[10] Ren A., Yao W., Zhu D. (2023). A Mitochondrion-Targeted Fluorescent Probe Based on ESIPT Phthalimide for the Detection of Hg ^2+^ with Large Stokes Shift. Analyst.

[11] Naskar R., Gharami S., Mandal S., Mondal T.K. (2022). A New Chromone-Based Fluorescent Probe for Ratiometric Detection of Pd^2+^. New J. Chem..

[12] Imran K., Pandey D., Kaur J., Naqvi S., Sharma A. (2023). An ESIPT Solvatochromic Fluorescent and Colorimetric Probe for Sensitive and Selective Detection of Copper Ions in Environmental Samples and Cell Lines. Analyst.

[13] Zhou Q., Wang H., Song P. (2023). Theoretical Study of the Direction of the Excited-State Intramolecular Proton Transfer of the HBS Molecule. New J. Chem..

[14] Li Y., Dahal D., Abeywickrama C.S., Pang Y. (2021). Progress in Tuning Emission of the Excited-State Intramolecular Proton Transfer (ESIPT)-Based Fluorescent Probes. ACS Omega.

[15] Chipem F.A.S., Krishnamoorthy G. (2013). Temperature Effect on Dual Fluorescence of 2-(2′-Hydroxyphenyl)Benzimidazole and Its Nitrogen Substituted Analogues. J. Phys. Chem. B.

[16] Cheng J., Liu D., Li W., Bao L., Han K. (2015). Comprehensive Studies on Excited-State Proton Transfer of a Series of 2-(2′-Hydroxyphenyl)Benzo-thiazole Derivatives: Synthesis, Optical Properties, and Theoretical Calculations. J. Phys. Chem. C.

[17] Li C., Yang Y., Ma C., Liu Y. (2016). Effect of Amino Group on the Excited-State Intramolecular Proton Transfer (ESIPT) Mechanisms of 2-(2′-Hydroxyphenyl)Benzoxazole and Its Amino Derivatives. RSC Adv..

[18] Abou-Zied O.K. (2013). Spectroscopy of Hydroxyphenyl Benzazoles in Solution and Human Serum Albumin: Detecting Flexibility, Specificity and High Affinity of the Warfarin Drug Binding Site. RSC Adv..

[19] Alarcos N., Gutiérrez M., Liras M., Sánchez F., Moreno M., Douhal A. (2015). Direct Observation of Breaking of the Intramolecular H-Bond, and Slowing down of the Proton Motion and Tuning Its Mechanism in an HBO Derivative. Phys. Chem. Chem. Phys..

[20] Chipem F.A.S., Dash N., Krishnamoorthy G. (2011). Role of Nitrogen Substitution in Phenyl Ring on Excited State Intramolecular Proton Transfer and Rotamerism of 2-(2′-Hydroxyphenyl)Benzimidazole: A Theoretical Study. J. Chem. Phys..

[21] Weigend F. (2006). Accurate Coulomb-Fitting Basis Sets for H to Rn. Phys. Chem. Chem. Phys..

[22] Manojai N., Daengngern R., Kerdpol K., Ngaojampa C., Kungwan N. (2017). Heteroatom Effect on Photophysical Properties of 2-(2′-Hydroxy-phenyl)Benzimidazole and Its Derivatives as Fluorescent Dyes: A TD-DFT Study. J. Lumin..

[23] Yang Y. (2020). The Effects of Amino Group Meta- and Para-Substitution on ESIPT Mechanisms of Amino 2-(2′-Hydroxyphenyl) Benzazole Derivatives. J. Lumin..

[24] Zhang Y., Shang C., Cao Y., Ma M., Sun C. (2022). Insights into the Photophysical Properties of 2-(2′-Hydroxyphenyl) Benzazoles Derivatives: Application of ESIPT Mechanism on UV Absorbers. Spectrochim. Acta A Mol. Biomol. Spectrosc..

[25] Zhao J., Chen J., Liu J., Hoffmann M.R. (2015). Competitive Excited-State Single or Double Proton Transfer Mechanisms for Bis-2,5-(2-Benzoxazolyl)-Hydroquinone and Its Derivatives. Phys. Chem. Chem. Phys..

[26] Lee H., Lee S., Han M.S. (2023). Turn-On Fluorescent pH Probes for Monitoring Alkaline pHs Using Bis[2-(2′-Hydroxyphenyl)Benzazole] Derivatives. Sensors.

[27] Goswami S., Das S., Aich K., Pakhira B., Panja S., Mukherjee S.K., Sarkar S. (2013). A Chemodosimeter for the Ratiometric Detection of Hydrazine Based on Return of ESIPT and Its Application in Live-Cell Imaging. Org. Lett..

[28] Liu Z.-Y., Hu J.-W., Chen C.-L., Chen Y.-A., Chen K.-Y., Chou P.-T. (2018). Correlation among Hydrogen Bond, Excited-State Intramolecular Proton-Transfer Kinetics and Thermodynamics for -OH Type Proton-Donor Molecules. J. Phys. Chem. C.

[29] Zhang H., Li Z., Liu J., Wang Y. (2022). Effect of Intermolecular Hydrogen Bonds on the Proton Transfer and Fluorescence Characteristics of 1′-Hydroxy-2′-Acetonaphthone. J. Mol. Liq..

[30] Wang J., Liu Q., Yang D. (2020). Theoretical Insights into Excited-State Hydrogen Bonding Effects and Intramolecular Proton Transfer (ESIPT) Mechanism for BTS System. Sci. Rep..

[31] Santos G.C., Rocha I.O., Stefanello F.S., Copetti J.P.P., Tisoco I., Martins M.A.P., Zanatta N., Frizzo C.P., Iglesias B.A., Bonacorso H.G. (2022). Investigating ESIPT and Donor-Acceptor Substituent Effects on the Photophysical and Electrochemical Properties of Fluorescent 3,5-Diaryl-Substituted 1-Phenyl-2-Pyrazolines. Spectrochim. Acta A Mol. Biomol. Spectrosc..

[32] Zhao G., Han K. (2008). Time-dependent Density Functional Theory Study on Hydrogen-bonded Intramolecular Charge-transfer Excited State of 4-dimethylamino-benzonitrile in Methanol. J. Comput. Chem..

[33] Zhao G.-J., Han K.-L. (2012). Hydrogen Bonding in the Electronic Excited State. Acc. Chem. Res..

[34] Zhang M., Guo Y., Feng X., Yu X., Jin X., Qiu L., Zhao G. (2019). Theoretical Modeling of the Hydrated Serotonin in Solution: Insight into Intermolecular Hydrogen Bonding Dynamics and Spectral Shift in the Electronic Excited States. J. Mol. Liq..

[35] Contreras-Garcia J., Johnson E.R., Keinan S., Chaudret R., Piquemal J.P., Beratan D.N., Yang W. (2011). NCIPLOT: A program for plotting non-covalent interaction regions. J. Chem. Theory Comput..

[36] Ren P., Sun C., Shi Y., Song P., Yang Y., Li Y. (2019). Global performance evaluation of solar cells using two models: From charge-transfer and recombination mechanisms to photoelectric properties. J. Mater. Chem. C.

[37] Dai Y., Zhang M., Zhang M., Sun L., Meng J., Song P. (2018). Intramolecular Hydrogen Bonding Promoted Excited State Double Proton Transfer Mechanism Based on a Typical Molecule: Porphycene. Spectrochim. Acta A Mol. Biomol. Spectrosc..

[38] Bader R.F.W. (1991). A Quantum Theory of Molecular Structure and Its Applications. Chem. Rev..

[39] Emamian S., Lu T., Kruse H., Emamian H. (2019). Exploring Nature and Predicting Strength of Hydrogen Bonds: A Correlation Analysis Between Atoms-in-Molecules Descriptors, Binding Energies, and Energy Components of Symmetry-Adapted Perturbation Theory. J. Comput. Chem..

[40] Luo X., Yang Y., Li Y. (2020). Theoretical Insights into ESIPT Mechanism of the Two Protons System BH-BA in Dichloromethane Solution. J. Mol. Liq..

[41] Chen C., Fang C. (2023). Fluorescence Modulation by Amines: Mechanistic Insights into Twisted Intramolecular Charge Transfer (TICT) and Beyond. Chemosensors.

[42] Fan Y., Wang F., Hou F., Wei L., Zhu G., Zhao D., Hu Q., Lei T., Yang L., Wang P. (2023). A Novel TICT-Based near-Infrared Fluorescent Probe for Light-up Sensing and Imaging of Human Serum Albumin in Real Samples. Chin. Chem. Lett..

[43] Miertuš S., Scrocco E., Tomasi J. (1981). Electrostatic interaction of a solute with a continuum: A direct utilizaion of AB initio molecular potentials for the prevision of solvent effects. Chem. Phys..

[44] Zhang Q., Yang Y., Liu Y. (2023). Theoretical Insights into Luminescence Mechanism of Naphthyridine-Based Thermally Activated Delayed Fluorescence Emitter with Aggregation-Induced Emission. Chem. Phys. Lett..

[45] Feller D. (1996). The role of databases in support computational chemistry calculations. J. Comput. Chem..

[46] Becke A. (1993). Density-functional thermochemistry. III. The role of exact exchange. J. Chem. Phys..

[47] Grimme S., Ehrlich S., Goerigk L. (2011). Effect of the Damping Function in Dispersion Corrected Density Functional Theory. J. Comput. Chem..

[48] Lin Y.D., Ke B.Y., Chang Y.J., Chou P.T., Liau K.L., Liu C.Y., Chow T.J. (2015). Pyridomethene-BF2 complex/phenothiazine hybrid sensitizer with high molar extinction coefficient for efficient, sensitized solar cells. J. Mater. Chem. A.

[49] Wang D., Zhou Q., Wei Q., Song P. (2023). Effects of π-Conjugation-Substitution on ESIPT Process for Oxazoline-Substituted Hydroxy-fluorenes. Chin. Phys. B.

[50] Lin S., Peng D., Yang W., Gu F.L., Lan Z. (2021). Theoretical Studies on Triplet-State Driven Dissociation of Formaldehyde by Quasi-Classical Molecular Dynamics Simulation on Machine-Learning Potential Energy Surface. J. Chem. Phys..

[51] Cohen A.J., Mori-Sánchez P., Yang W. (2008). Insights into Current Limitations of Density Functional Theory. Science.

[52] Zhang P., Yang W. (2023). Toward a General Neural Network Force Field for Protein Simulations: Refining the Intramolecular Interaction in Protein. J. Chem. Phys..

[53] Yang Y., Zhao J., Li Y. (2016). Theoretical Study of the ESIPT Process for a New Natural Product Quercetin. Sci. Rep..

[54] Ding Z., Ji S., Zhao J., Zheng D. (2022). Combination of Theoretical Calculation and Experiment to Study the Excited State Proton Transfer Behavior of Trifluoroacetamidoanthraquinone with Different Substitution Positions. J. Mol. Struct..

[55] Li Q., Wan Y., Zhou Q., Li Y., Li B., Zhu L., Wan Y., Yin H., Shi Y. (2022). Exploring the Effect of Nitrile Substituent Position on Fluorescence Quantum Yield of ESIPT-Based Oxazoline Derivatives: A TDDFT Investigation. Spectrochim. Acta A Mol. Biomol. Spectrosc..

[56] Alecu I.M., Zheng J., Zhao Y., Truhlar D.G. (2010). Computational Thermochemistry: Scale Factor Databases and Scale Factors for Vibrational Frequencies Obtained from Electronic Model Chemistries. J. Chem. Theory. Comput..

[57] Schlegel H.B. (1982). Optimization of equilibrium geometries and transition structures. J. Comput. Chem..

[58] Fukui K., Yonezawa T., Shingu H. (1952). A Molecular Orbital Theory of Reactivity in Aromatic Hydrocarbons. J. Chem. Phys..

[59] Fukui K., Yonezawa T., Nagata C., Shingu H. (1954). Molecular Orbital Theory of Orientation in Aromatic, Heteroaromatic, and Other Conjugated Molecules. J. Chem. Phys..

[60] Lu T., Chen F. (2012). Multiwfn: A multifunctional wavefunction analyzer. J. Comput. Chem..

[61] Humphrey W., Dalke A., Schulten K. (1996). VMD: Visual molecular dynamics. J. Mol. Graph..

[62] Frisch M.J., Trucks G.W., Schlegel H.B., Scuseria G.E., Robb M.A., Cheeseman J.R., Scalmani G., Barone V., Petersson G.A., Nakatsuji H. (2016). Gaussian 16.

[63] Miehlich B., Savin A., Stoll H., Preuss H. (1989). Results obtained with the correlation energy density functionals of becke and Lee, Yang and Parr. Chem. Phys. Lett..

[64] Yanai T., Tew D.P., Handy N.C. (2004). A new hybrid exchange–correlation functional using the Coulomb-attenuating method (Cam-B3LYP). Chem. Phys. Lett..

[65] Freeman F. (2014). Mechanism of the cysteine sulfenic acid *O*-sulfenylation of 1,3-cyclohexanedione. Chem. Commun..

[66] Park K., Son H.-J., Choe J.-I. (2014). mPW1PW91 study for conformational isomers of methylene bridge-monosubstituted tetramethoxycalix[4]arenes. J. Ind. Eng. Chem..

