# Supplementary material for: Screening the Optimal Probe by Expounding the ESIPT Mechanism and Photophysical Properties in Bis-HBX with Multimodal Substitutions
Source: Molecules. 2024 Jun 6;29(11):2692. doi: 10.3390/molecules29112692 (PMC11173473; doi:10.3390/molecules29112692)
Supplement: Supplementary file 1 [file molecules-29-02692-s001.zip › molecules-3032236-supplementary.pdf]

Screening the Optimal Probe by Expounding the ESIPT Mechanism and Photophysical Properties in Bis-HBX with Multimodal Substitutions

Min Yang [1], Hongyan Mu [1], Jiaan Gao [1], Qi Zhen [2], Xiaonan Wang [1], Xiaotong Guan [1], Hui Li [1,*] and Bo Li [3,*]

[1] Jilin Key Laboratory of Solid-State Laser Technology and Application, School of Physics, Changchun University of Science and Technology, Changchun 130022, China; 18404361217@163.com (M.Y.); xuemuafm@163.com (H.M.); gja13622147194@163.com (J.G.); wang18338564758@163.com (X.W.); mianhuatang7657@163.com (X.G.)

[2] School of Civil Engineering, Changchun Institute of Technology, Changchun 130012, China; zhenqi18@mails.jlu.edu.cn

[3] State Key Laboratory of High Power Semiconductor Lasers, School of Physics, Changchun University of Science and Technology, Changchun 130022, China

* Correspondence: huili@cust.edu.cn (H.L.); libo@cust.edu.cn (B.L.); Tel./Fax: +86-431-85582465 (H.L.)

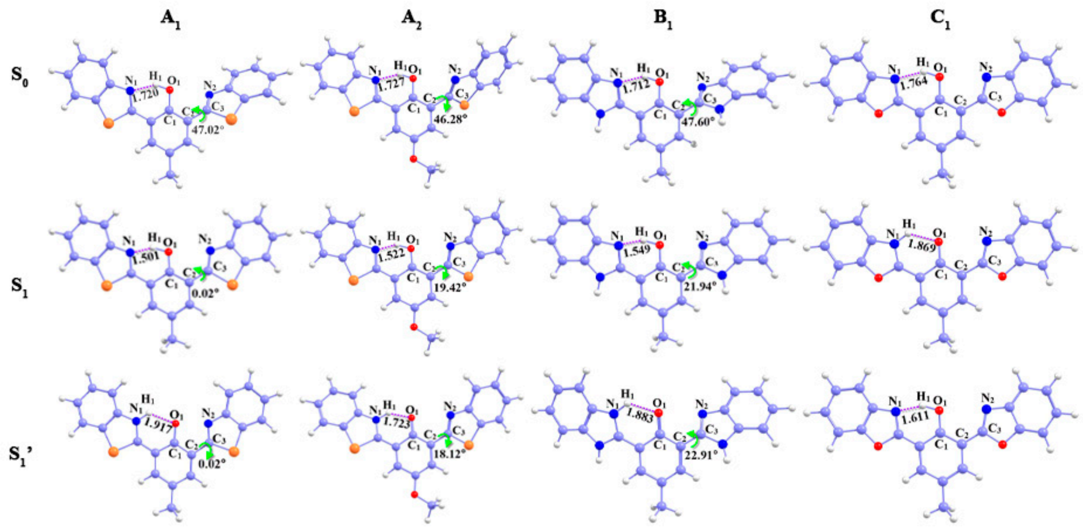

Figure S1. Optimized geometry of bis-HBX in ground state.

Table S1 Experimental and theoretical values of absorption and fluorescence peaks of bis-HBX.

	Exp.-Abs.	Exp.-Flu.	Abs.	Flu.-S_1	Flu.-S_1’	τ
A_1	290/373	503	295/368	463	622	5.1121
A_2	293/400	535	302/399	481	633	5.1027
B_1	280/360	413	283/356	432	566	4.6438
C_1	290/358	490	288/365	419	541	2.4009

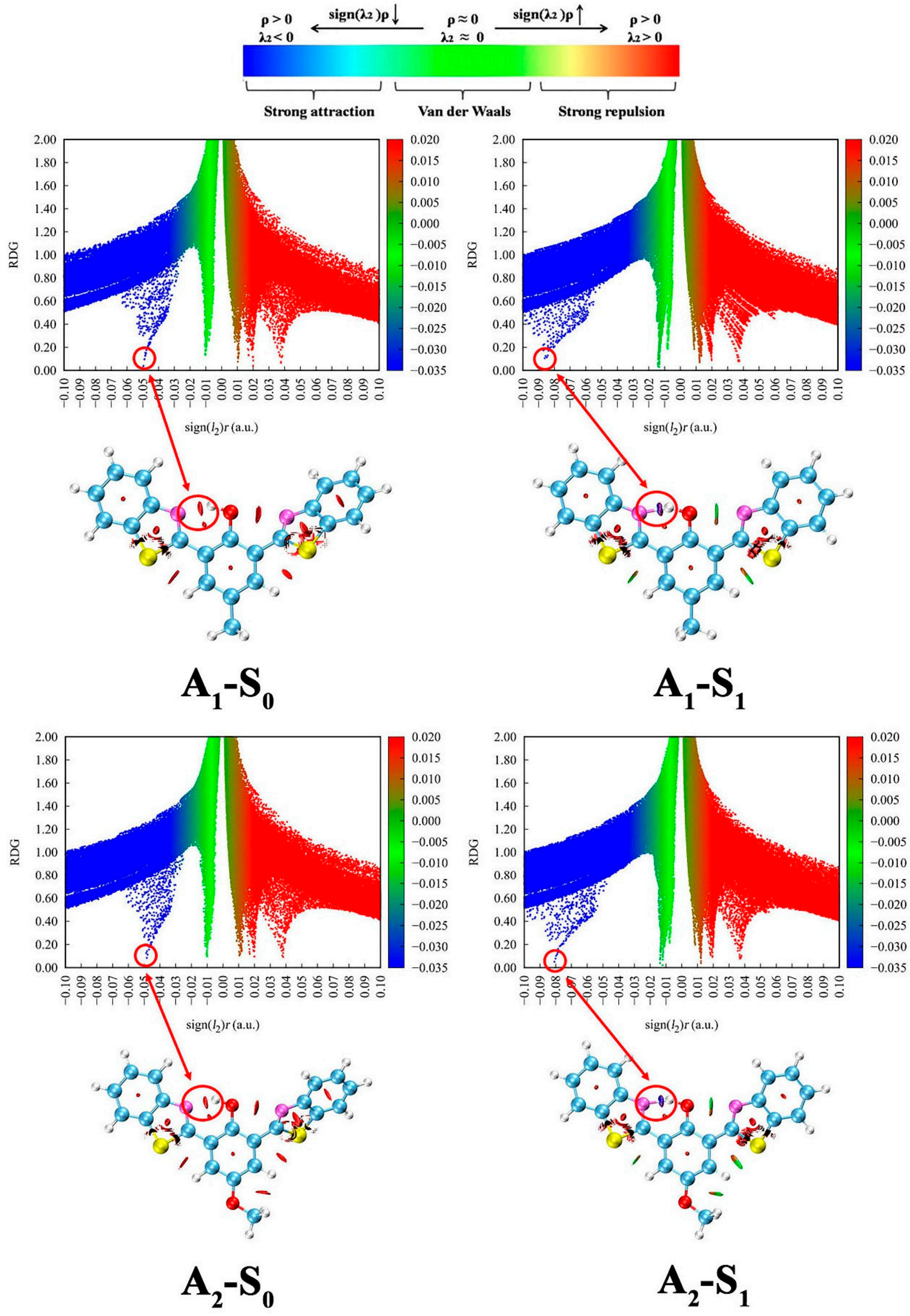

Figure S2. Sign(I_2)r scatter plots versus reduced density gradient (RDG) in A_1 and A_2 in the different states.

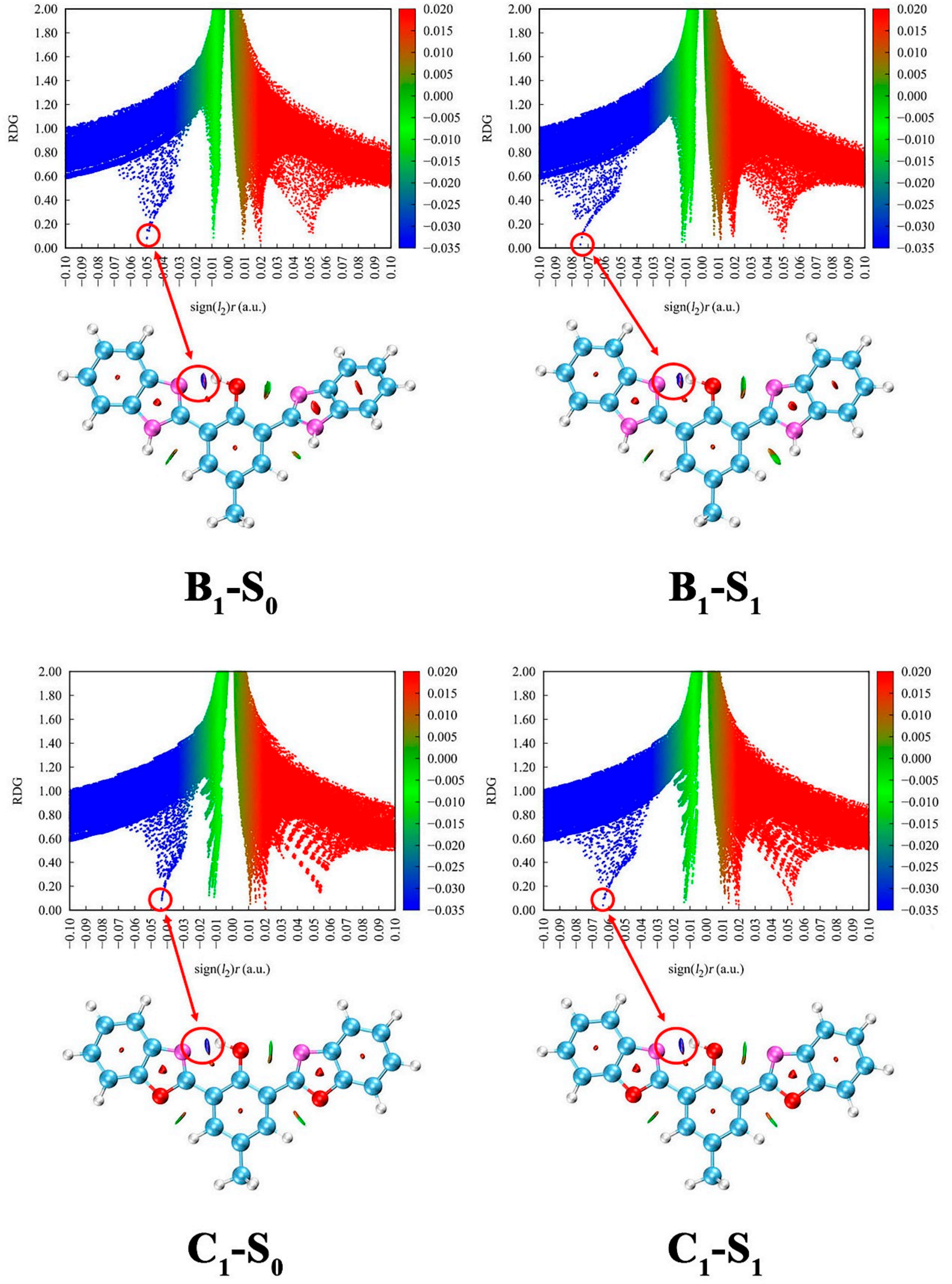

Figure S3. Sign(I_2)r scatter plots versus reduced density gradient (RDG) in B_1 and C_1 in the different states.

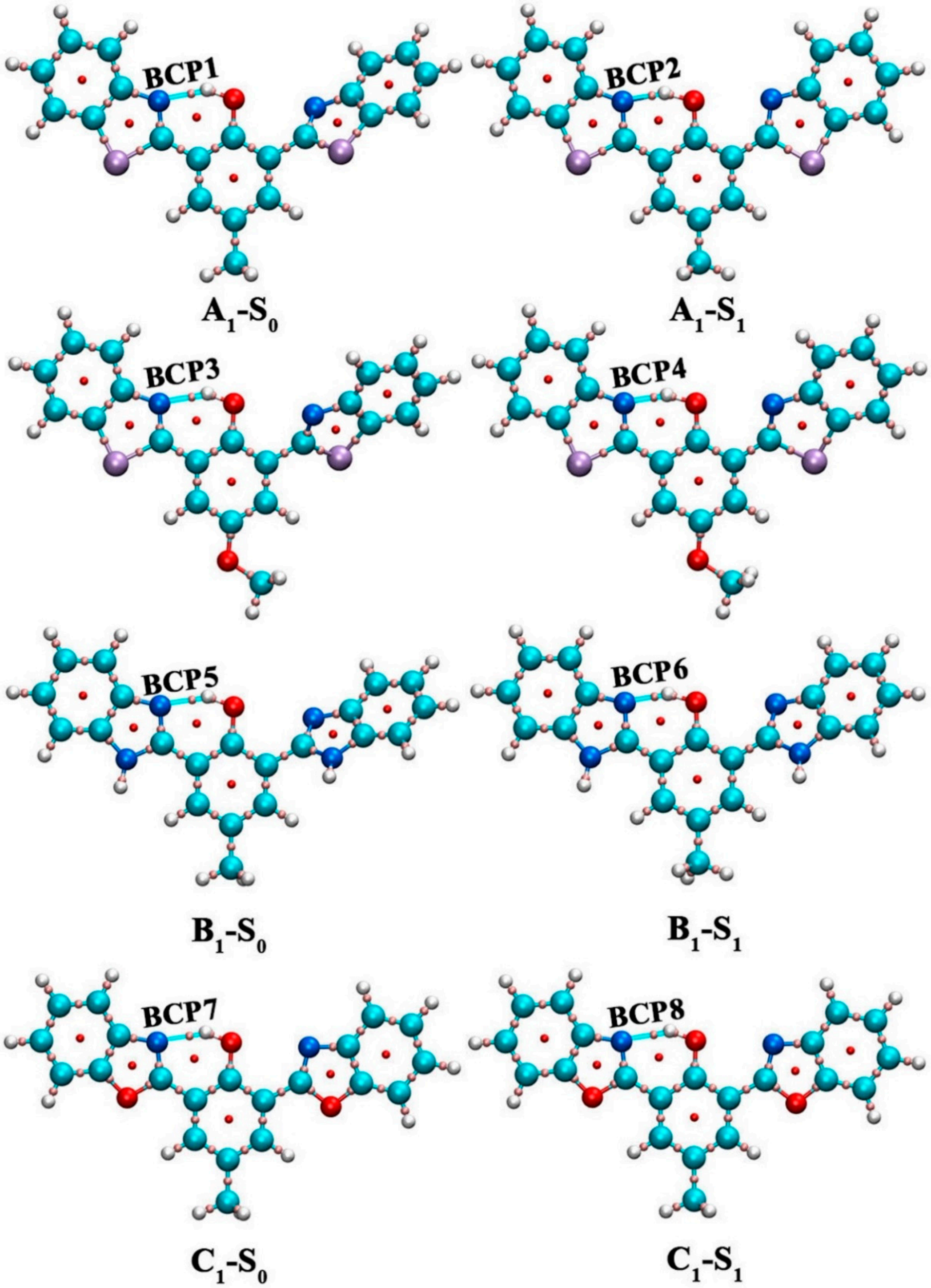

Figure S4. Topological diagrams of bis-HBX. The points in O_1−H_1...N_1 regions represented bond critical points (BCPs) involved in IHBs, and the cyan lines represented bond paths.

Ground state optimized Cartesian coordinates

A_1

C	-5.37410000	5.53940000	-0.36760000
C	-4.43210000	4.39060000	-0.26110000
C	-4.89900000	3.14300000	-0.17040000
C	-6.33630000	2.95500000	-0.17720000
C	-7.17970000	3.96830000	-0.27020000
C	-6.69500000	5.33400000	-0.37200000
N	-8.55480000	3.58920000	-0.25480000
C	-8.71010000	2.32000000	-0.15270000
S	-7.17910000	1.45400000	-0.07030000
C	-11.16360000	2.32670000	-0.14490000
C	-10.01550000	1.64070000	-0.10260000
C	-10.04250000	0.17820000	0.00120000
C	-11.20110000	-0.47270000	0.05560000
C	-12.45710000	0.26790000	0.00600000
C	-12.45080000	1.59560000	-0.08990000
C	-16.95900000	5.63710000	-0.36660000
C	-15.64600000	5.38900000	-0.36240000
C	-15.20650000	4.00730000	-0.26110000
C	-16.08230000	3.02190000	-0.17630000
C	-17.51290000	3.25690000	-0.17960000
C	-17.93890000	4.51930000	-0.27040000
S	-15.29040000	1.49390000	-0.06740000
C	-13.73230000	2.30850000	-0.13950000
N	-13.84170000	3.58330000	-0.23870000
O	-11.14990000	3.72350000	-0.23530000
C	-11.21610000	-1.96910000	0.16710000
H	-5.01630000	6.47070000	-0.43660000
H	-3.44560000	4.55430000	-0.25770000
H	-4.27690000	2.36320000	-0.09980000
H	-7.33310000	6.10060000	-0.44350000
H	-9.18370000	-0.33330000	0.03150000
H	-13.32680000	-0.22420000	0.04420000
H	-17.28580000	6.57970000	-0.43520000
H	-14.98290000	6.13470000	-0.42760000
H	-18.16040000	2.49740000	-0.11610000
H	-18.91960000	4.71500000	-0.27390000
H	-12.07710000	4.06830000	-0.24860000
H	-12.16190000	-2.29220000	0.19600000
H	-10.74390000	-2.24620000	1.00390000
H	-10.75230000	-2.36740000	-0.62440000

A_2

C	5.87097000	-2.92818500	-0.76143500
C	6.60207100	-2.17802900	0.17955100
C	6.04408500	-1.05904600	0.79605500
C	4.73551500	-0.70203800	0.45465600
C	3.98859300	-1.45125000	-0.49115900
C	4.56982300	-2.57333800	-1.10181800
N	2.70947700	-0.98744400	-0.74262600
C	2.43620400	0.06758100	-0.04422700
S	3.75425000	0.62076100	1.04358900
C	-0.06938200	0.14764000	-0.03313600
C	1.17133000	0.82209700	-0.08155700
C	1.21113400	2.22284800	-0.15051800
C	0.03279300	2.97451200	-0.19126900
C	-1.19368600	2.31763700	-0.15843200
C	-1.26981000	0.91904900	-0.07740500
C	-5.81486400	-3.04708900	0.22938000
C	-4.44683700	-2.79482400	0.21195300
C	-4.00716500	-1.46702400	0.10735100
C	-4.95133300	-0.41567000	0.02211300
C	-6.32546700	-0.66946800	0.03947500
C	-6.74595400	-1.99502900	0.14404100
S	-4.11233600	1.11854000	-0.09920100
C	-2.56245000	0.24209900	-0.02591500
N	-2.68721100	-1.05821700	0.07755400
O	-0.07435100	-1.18814700	0.08287500
O	-0.01847600	4.34116300	-0.27254000
C	1.20802600	5.06197900	-0.29607600
H	6.33058700	-3.79538600	-1.22586000
H	7.61627400	-2.47367700	0.43101300
H	6.61073300	-0.48358600	1.52106500
H	3.99225900	-3.14067200	-1.82407700
H	2.17898600	2.70636900	-0.19681400
H	-2.09261000	2.92431200	-0.19553900
H	-6.17043600	-4.06960100	0.30966600
H	-3.71836000	-3.59628100	0.27649300
H	-7.04582500	0.13935200	-0.02640400
H	-7.80874100	-2.21605800	0.15941400
H	-1.01659700	-1.49611900	0.11378000
H	0.93218900	6.11573200	-0.35104000
H	1.81187300	4.79877500	-1.17459100
H	1.79421500	4.88682100	0.61575300

B_1

C	6.25386200	-2.20275200	0.44309600
C	6.70896400	-1.28833900	-0.53195600
C	5.88650900	-0.27163200	-1.01766800
C	4.59363900	-0.20581300	-0.49281300
C	4.11700800	-1.11996900	0.48097000
C	4.96199600	-2.13118900	0.95911100
N	2.80270600	-0.83236100	0.81352100
C	2.47733300	0.21003900	0.08252100
N	3.52676000	0.64453600	-0.71771000
C	-0.03799100	0.25392300	0.04026100
C	1.20031500	0.94189300	0.09403500
C	1.21045300	2.33834900	0.14642300
C	0.03320300	3.10198000	0.16308900
C	-1.17600700	2.41586700	0.12565900
C	-1.24196200	1.01090300	0.06461500
C	-6.06011200	-2.48134300	-0.20033100
C	-4.66703800	-2.50803600	-0.20707900
C	-3.99088400	-1.28537600	-0.11210500
C	-4.71972600	-0.07600300	-0.01446700
C	-6.11479200	-0.04080200	-0.00664800
C	-6.77297700	-1.26764800	-0.10148300
N	-3.75533500	0.91707300	0.06047300
C	-2.51791700	0.30880100	0.01103800
N	-2.63466300	-1.00969200	-0.09373000
O	-0.02772300	-1.07889100	-0.06073500
C	0.09374300	4.60977400	0.24511700
H	6.92847600	-2.97803100	0.79405900
H	7.72239800	-1.37748700	-0.91192100
H	6.23994700	0.43162700	-1.76596400
H	4.60431200	-2.83215000	1.70625300
H	3.46169200	1.35526800	-1.43005300
H	2.16957600	2.84741100	0.20872100
H	-2.09541800	2.99603200	0.14082200
H	-6.61242100	-3.41324200	-0.27198100
H	-4.11353700	-3.43815100	-0.28179800
H	-6.66543200	0.89178500	0.06945900
H	-7.85844300	-1.28797000	-0.09887300
H	-3.93007000	1.90422100	0.15611700
H	-0.96905800	-1.40429000	-0.09839300
H	-0.90313500	5.05483500	0.17173200
H	0.70550300	5.02958100	-0.56163300
H	0.53540800	4.93902300	1.19316000

C_1

C	-6.27333600	-2.21678400	0.00068500
C	-4.88953300	-2.40713600	0.00064500
C	-4.08913200	-1.26191900	0.00034300
C	-4.68093800	0.00928000	0.00009400
C	-6.04966800	0.22468000	0.00011700
C	-6.84322100	-0.92872300	0.00042300
O	-3.66753000	0.94321300	-0.00018900
C	-2.51145400	0.20097600	-0.00005400
N	-2.70610600	-1.09469800	0.00023200
C	6.32797600	-2.18936100	-0.00100500
C	6.85370600	-0.88163600	0.00155500
C	6.02162000	0.24400900	0.00277200
C	4.65941800	-0.01735300	0.00129200
C	4.10995100	-1.30792400	-0.00123400
C	4.95162700	-2.42496000	-0.00244200
N	2.72175200	-1.20721800	-0.00212600
C	2.47701000	0.06989300	-0.00023000
O	3.61721600	0.87204900	0.00197600
C	-0.04088600	0.10848700	-0.00010800
C	1.20577700	0.78993300	-0.00028800
C	1.21384000	2.19279000	-0.00051500
C	0.04291100	2.96113600	-0.00067300
C	-1.17419700	2.28766300	-0.00055700
C	-1.23420800	0.88282500	-0.00025500
O	-0.06051900	-1.22748700	0.00028100
C	0.11691100	4.47073500	-0.00098300
H	-6.92854400	-3.08229600	0.00092300
H	-4.44707200	-3.39735300	0.00084700
H	-6.47511100	1.22180300	-0.00008800
H	-7.92377100	-0.82602500	0.00045500
H	7.01305000	-3.03173100	-0.00187300
H	7.93045100	-0.74240300	0.00261700
H	6.41470200	1.25461000	0.00474900
H	4.54036100	-3.42870000	-0.00440900
H	2.17364900	2.69805500	-0.00061500
H	-2.10527400	2.84526300	-0.00069900
H	-0.99946700	-1.54188100	0.00049500
H	-0.88109700	4.91820900	-0.00111100
H	0.65013800	4.84182500	-0.88380100
H	0.65007200	4.84217800	0.88172600
